# Stent Grafting for a Saccular Aneurysm of Cryopreserved Homograft after Implantation for Infectious Abdominal Aortic Aneurysm

**DOI:** 10.3400/avd.cr.26-00051

**Published:** 2026-07-29

**Authors:** Yosuke Yanase, Yuka Matsuda, Junichi Sakata, Masanori Nakamura

**Affiliations:** Department of Cardiovascular Surgery, Sapporo City General Hospital, Sapporo, Hokkaido, Japan

**Keywords:** homograft, stent graft, endovascular therapy

## Abstract

An 82-year-old man underwent surgical repair of an infectious abdominal aortic aneurysm using a cryopreserved homograft. Twelve years later, a pseudoaneurysm at the right iliac anastomosis was treated with a stent-graft limb. Sixteen years after the initial surgery, a saccular aneurysm developed in the mid-portion of the homograft without signs of infection. Endovascular aortic repair using an AFX2 stent graft (Endologix, Irvine, CA, USA) was performed to cover the entire graft. Postoperative CT showed no endoleaks or aneurysm enlargement. Although homografts are considered infection-resistant, late degenerative complications may occur, requiring lifelong follow-up.

## Introduction

Cryopreserved homografts are generally considered resistant to infection and have been used for the treatment of infectious aneurysms.^1)^ However, because their use is relatively limited, reports on long-term outcomes and complications are scarce. Herein, we report a case of a saccular aneurysm that developed in the late phase after homograft implantation for an infectious abdominal aortic aneurysm, likely due to graft degeneration.

## Case Report

An 82-year-old man had undergone abdominal aortic replacement with a cryopreserved homograft for an infectious abdominal aortic aneurysm 16 years previously (**Fig. 1**).^2)^ The proximal anastomosis was performed below the renal arteries, and the distal anastomoses were made to the right external iliac artery and the left common iliac artery. The inferior mesenteric artery was also reconstructed, and the homograft was wrapped with the omentum. The patient had been followed regularly without any evidence of recurrent infection.

**Fig. 1:**
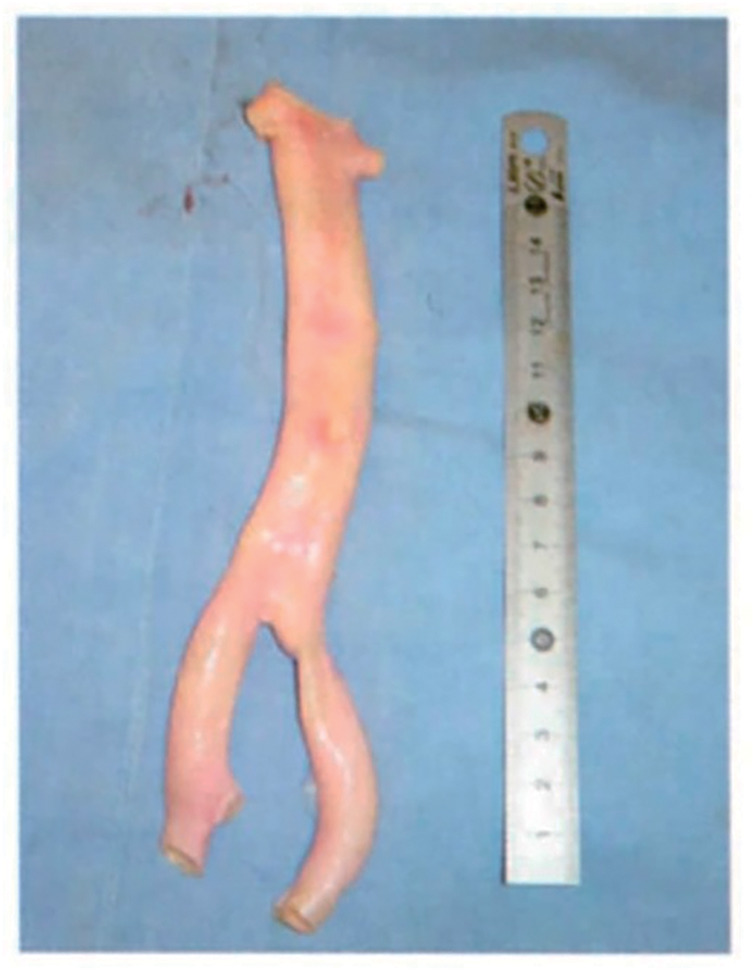
Homograft. Reproduced from Acta Medica Nosocomii Sapporo 2010; 69: 267–71.^2)^

At 12 years after the initial surgery, a pseudoaneurysm developed at the anastomotic site of the right external iliac artery and was treated with endovascular placement of a stent-graft limb. During follow-up imaging this year, a new saccular aneurysm was identified in the mid-portion of the homograft (**Fig. 2**), and the patient was admitted for treatment.

**Fig. 2:**
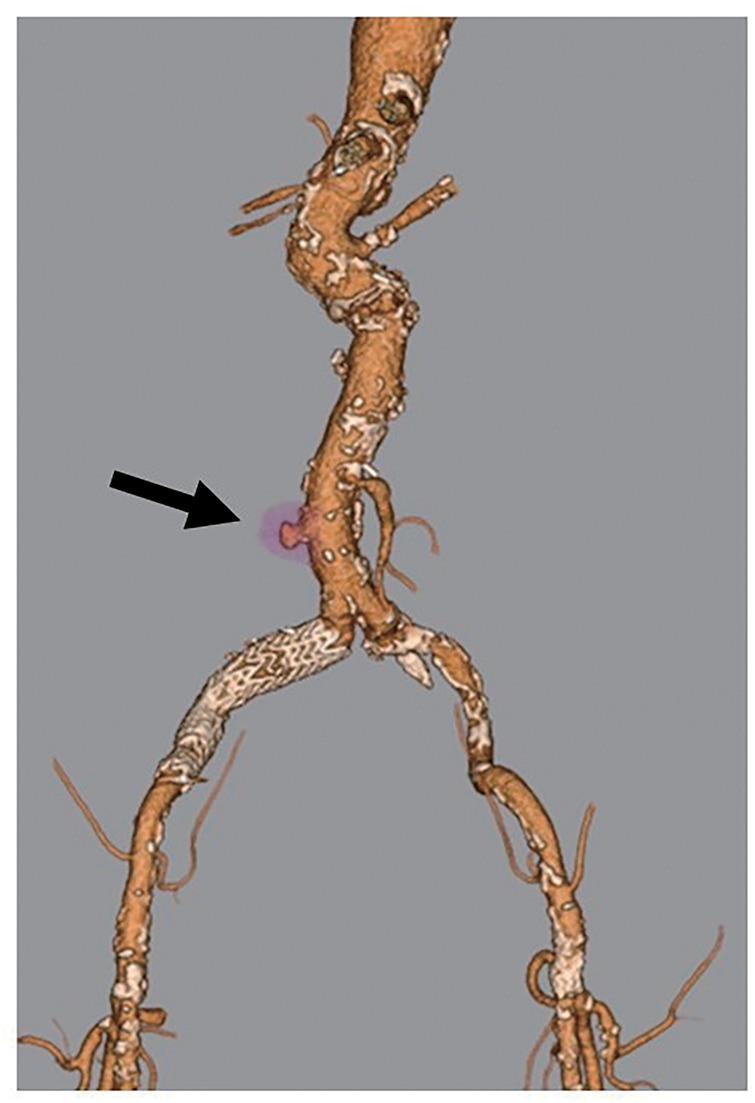
Preoperative eCT. Black arrow: Saccular aneurysm of the homograft. eCT: contrast-enhanced computed tomography

On admission, his body temperature was 36.1°C, blood pressure was 142/77 mmHg, and heart rate was 73 beats/min. Laboratory data showed a white blood cell count of 4200/µL and a C-reactive protein level of 0.32 mg/dL. The ankle–brachial index was 0.93 on the right and 0.78 on the left. Contrast-enhanced CT revealed a saccular aneurysm in the mid-portion of the homograft, measuring 36 mm in maximum diameter (height: 15 mm; width: 18 mm).

Given the absence of signs of infection recurrence and the patient’s high surgical risk due to advanced age, chronic renal failure requiring dialysis, and prior laparotomy, we selected endovascular treatment. Under general anesthesia, endovascular aortic repair (EVAR) was performed. Because the reconstructed inferior mesenteric artery was relatively large (6 mm in diameter), coil embolization was performed prior to stent-graft deployment. An AFX2 stent graft system (Endologix, Irvine, CA, USA) was selected. A proximal VELA cuff (Endologix) was additionally deployed to extend coverage up to the proximal portion of the initial anastomotic site. For left common iliac artery stenosis, balloon angioplasty was performed followed by placement of a S.M.A.R.T. CONTROL stent (8 × 60 mm; Cordis, Miami Lakes, FL, USA), extending from the stent-graft limb to the external iliac artery. Completion angiography confirmed the absence of endoleaks.

Postoperative CT showed no endoleaks (**Fig. 3**). The postoperative course was uneventful, and the patient was discharged on postoperative day 8. At 6 months of follow-up, CT demonstrated no aneurysm enlargement or new aneurysm formation.

**Fig. 3:**
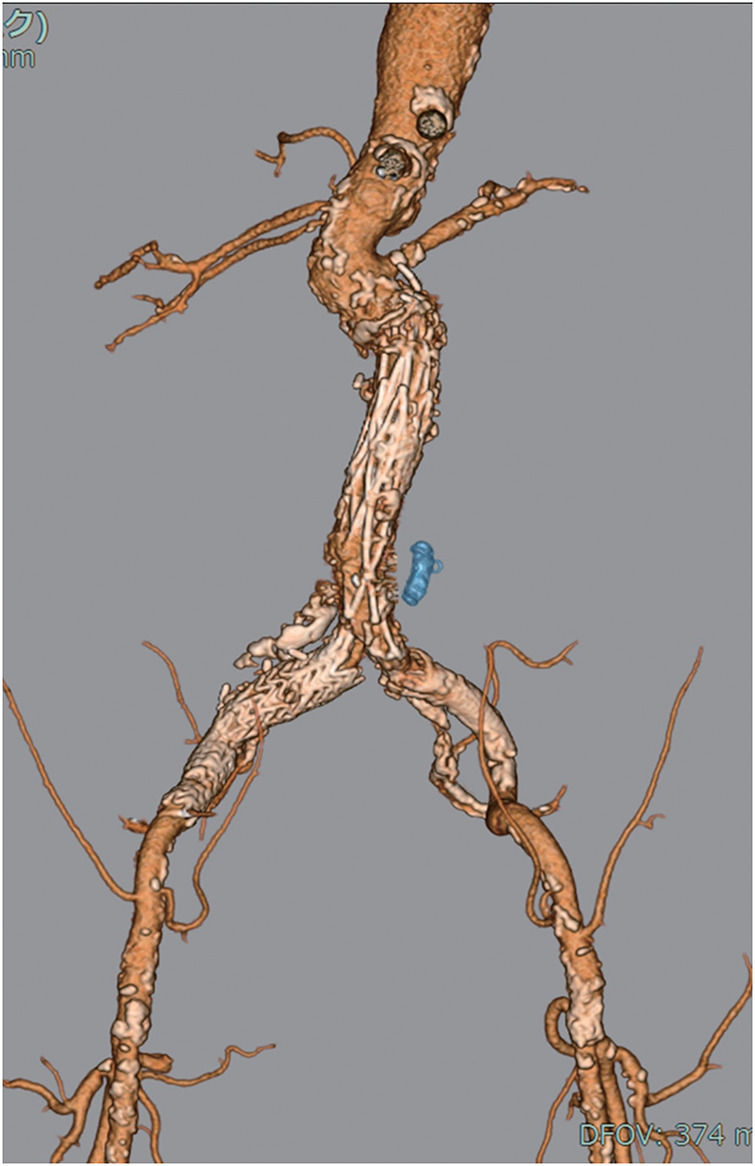
Postoperative eCT. No endoleak was observed. eCT: contrast-enhanced computed tomography

## Discussion

Infectious aortic aneurysms are rare but potentially life-threatening due to their rapid progression and high risk of rupture.^3)^ Antibiotic therapy alone is often insufficient, and surgical intervention is frequently required. Surgical options include aneurysm exclusion with extra-anatomic bypass,^4)^ endovascular repair,^5)^ and in situ reconstruction following aneurysm resection.^1)^

In the present case, in situ reconstruction using a cryopreserved homograft was selected at the initial surgery with the expectation of both radical cure and resistance to infection. More than 10 years after surgery, no recurrence of infection has been observed, suggesting that infection control was successful.

However, because cryopreserved homografts are not widely available, the number of reported cases is limited, and data regarding long-term outcomes remain scarce.^6,7)^ Available reports indicate that late complications include aneurysmal degeneration and rupture due to graft deterioration. As previously reported, pseudoaneurysm formation following homograft replacement has been described at a relatively high rate, with Oechslin et al. reporting its occurrence in 22 of 30 cases (73%), particularly in the aortic root,^8)^ raising concerns regarding durability.

On the other hand, a meta-analysis by Antonopoulos et al. demonstrated that in patients treated with homografts for infected abdominal aortic and iliac artery disease, homograft-related complications were relatively infrequent, including peri-anastomotic rupture/allograft disruption in 5.9%, aneurysmal degeneration/allograft dilatation in 4.99%, and pseudoaneurysm in 3.11%, suggesting an acceptable overall complication profile.^9)^

Histopathological studies of degenerated homografts have shown structural changes such as disruption of elastic fibers and myxoid degeneration of the vessel wall. Proposed mechanisms of degeneration include cryopreservation-related vascular injury, immune-mediated tissue damage, intraoperative handling, and mechanical injury from clamping.^9)^

Although pathological confirmation was not available in this case, the development of a saccular aneurysm more than 10 years after surgery, in the absence of clinical or laboratory evidence of infection, suggests graft degeneration as the most likely cause. However, previous reports have described disruption related to ligated collateral branches of the homograft,^7)^ and this possibility cannot be excluded in our case. Therefore, although the lesion appeared as a saccular aneurysm, it was not possible to definitively distinguish between a true aneurysm and a pseudoaneurysm in our case.

While cryopreserved homografts have been considered useful for treating infectious aneurysms, rifampin-soaked Dacron grafts have also been reported to provide comparable outcomes.^10)^ It has become evident that homografts may not be as resistant to infection as initially expected and are associated with potential long-term complications such as degeneration. Therefore, they are not necessarily the first-line option, and careful lifelong surveillance is required when they are used.

In this patient, endovascular therapy was selected due to high surgical risk, including advanced age, chronic renal failure, and a history of laparotomy with omental coverage. Given the saccular morphology and relatively narrow terminal aorta, an AFX2 stent graft was chosen. Considering the previous occurrence of a pseudoaneurysm at the anastomotic site, the entire length of the homograft was relined to prevent recurrence. Postoperative imaging demonstrated no endoleaks, and follow-up CT at 6 months showed no aneurysm enlargement, indicating that this strategy was appropriate.

## Conclusion

A saccular aneurysm arising from a cryopreserved homograft was successfully treated with endovascular therapy. Given the uncertain long-term durability of homografts, lifelong follow-up is essential.
